# Comparative Uncertainty Estimation in Neural Network Analysis of Wearable Sensor Signal for Cough and Fall Detection

**DOI:** 10.3390/s26134081

**Published:** 2026-06-27

**Authors:** Minh Long Hoang, Cesare Svelto, Paolo Ciampolini, Guido Matrella, Giovanni Chiorboli

**Affiliations:** 1 Department of Engineering and Architecture, University of Parma, 43124 Parma, Italy; paolo.ciampolini@unipr.it (P.C.); guido.matrella@unipr.it (G.M.); giovanni.chiorboli@unipr.it (G.C.); 2 Department of Electronics, Information and Bioengineering, Politecnico di Milano, 20133 Milano, Italy; cesare.svelto@polimi.it

**Keywords:** Predictive and Uncertainty Assessment Framework, bootstrap, deep learning, human activity recognition, Monte Carlo Dropout, uncertainty estimation

## Abstract

This paper presents research on a Predictive and Uncertainty Assessment Framework (PUAF), providing a comparative analysis of two prominent methods, Monte Carlo (MC) Dropout and Bootstrap-based models, used in uncertainty estimation techniques of Neural Network predictions of human activity recognition using accelerometer data. Unlike traditional studies that optimize classification accuracy, this work emphasizes uncertainty quantification to enhance model reliability, particularly for critical health-related activities. Among the five activity classes of Sit, Sleep, Walk, Cough and Fall, this work concentrates on the Cough and Fall cases. The study exploits acceleration data from a wearable device positioned on the user’s chest, with features derived from three-axis motion measurements. Synthetic datasets are generated by systematically introducing noise variations, added to the original dataset across all axes, to assess robustness under real-world conditions. Each uncertainty estimation method estimates the probabilities for the five different classes along with the corresponding 95% confidence intervals to quantify the prediction uncertainty. A detailed evaluation is conducted by analyzing the average width of these confidence intervals across different noise levels, identifying the most reliable feature and model combination. Both the MC Dropout and Bootstrap enhance model robustness and uncertainty awareness under noisy sensor conditions. The MC Dropout provides sharper and more sensitive uncertainty estimates, while the Bootstrap yields more stable and better-calibrated predictions. The evaluation using the proposed PUAF demonstrates that each method offers distinct advantages, highlighting the importance of uncertainty quantification for reliable wearable-based HAR systems.

## 1. Introduction

Human activity recognition (HAR) [1,2] using wearable sensor data has gained significant attention in recent years, particularly for health-monitoring applications. Wearable devices equipped with inertial measurement units (IMUs) [3,4,5,6,7], including accelerometers [8,9,10,11], provide continuous monitoring of human motion and enable automated recognition of activities. Among the various applications of HAR, detecting critical activities, such as Coughing and Falling, is particularly important for patient safety, early disease diagnosis, and elderly care. However, the reliability of the available predictive models remains a significant challenge, especially in real-world conditions where sensor data is subject to noise and variations.

Most existing research in HAR has primarily focused on optimizing classification accuracy with Machine Learning [12] and Deep Neural Networks (DNNs) [13,14,15,16,17,18,19,20], which usually neglect the quantification of uncertainty in model predictions. However, uncertainty estimation in DNN predictions has emerged as a crucial aspect of reliable decision-making in HAR. Traditional Neural Network (NN) models often provide point estimates for class predictions without quantifying the confidence associated with these predictions. This limitation can lead to misclassifications, particularly when dealing with noisy or incomplete data. To address this issue, uncertainty estimation techniques [21] have been utilized to measure the reliability of model outputs, thereby improving interpretability and robustness. Article [22] presented a unified framework that combines automatic feature extraction, classification, and uncertainty estimation for HAR. The authors quantified both the epistemic and aleatoric uncertainties, demonstrating how each responds to different sources of uncertainty in wearable sensor data. Other research [23] addressed the challenges of context-dependent activity characteristics and unknown contexts in wearable computing. The authors developed a context-aware mixture of deep models, coupled with uncertainty quantification based on maximum entropy, to improve HAR performance and facilitate unknown context discovery.

In this paper, the system works based on a Monte Carlo (MC) Dropout [24] and Bootstrap model [25] to assess uncertainty in predictions related to Cough and Fall activities using a chest-worn device with accelerometer data [26]. The comparison of these models provides a novel and systematic comparison of two fundamentally different uncertainty quantification paradigms: stochastic regularization via MC Dropout [27] and ensemble variance modeling through the Bootstrapping technique [28]. MC Dropout [29] introduces stochasticity in a network by randomly dropping units during both training and inference, approximating the Bayesian inference and yielding probabilistic predictions. In contrast, Bootstrap methods [30] rely on resampling techniques to create multiple models trained on different subsets of the data, thereby capturing variance in the predictions. Both methods offer distinct advantages in terms of computational complexity and reliability, making their comparative analysis essential for practical applications of wearable-based HAR. By comparing these two uncertainty estimation methods, the paper aims to enhance model interpretability and robustness, particularly in the presence of noisy and constrained data.

Publication [31] discusses brain–computer interfaces, applying the MC Dropout method to deep neural models for motion imagery classification. The approach not only improves classification accuracy but also provides uncertainty estimation. Another work [32] compares MC Dropout and Bootstrap aggregation techniques for uncertainty estimation in deep learning models for radiation therapy. The methodologies and findings offer valuable insights applicable to HAR, particularly for understanding model confidence and reliability. However, these works do not explicitly explore how their models behave under different noise levels, making their applicability to wearable HAR systems less robust. Additionally, work [33] emphasizes the personalization of HAR models through Bootstrap methods combined with active learning. While personalization is beneficial, the study does not address the robustness of these models under noisy conditions, which is a key aspect of the concerned field.

**Figure 1:**
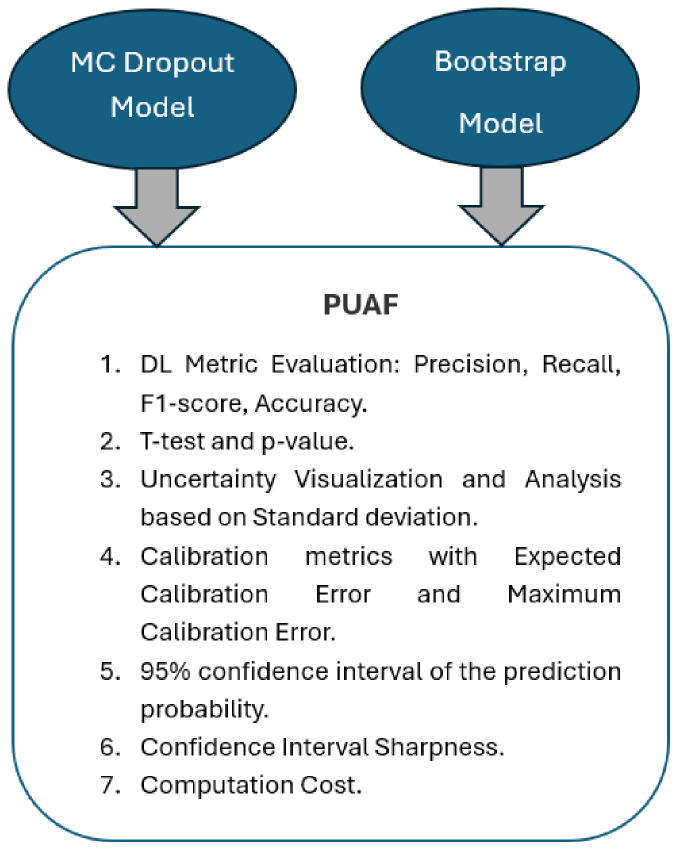
PUAF system for comparison between uncertainty analysis methods.

Based on the above considerations, this study provides a Predictive and Uncertainty Assessment Framework (PUAF) for the two uncertainty estimation methods for classifying Cough and Fall activities based on accelerometer data collected from a wearable device on the chest in the presence of noise. Overall, there are five activities which are studied: Cough, Fall, Sit down, Sleep, and Walk. Noises are systematically introduced into the accelerometer readings across all three axes to simulate real-world scenarios, generating synthetic datasets derived from actual measurements. The uncertainty of class predictions is quantified using 95% confidence intervals, and the robustness of each method is assessed by analyzing the average width of these intervals versus the varying noise levels. The PUAF involves seven main procedures, as shown in Figure 1.

Additionally, we explore augmentation techniques based on MC Dropout and Bootstrap to enhance NN performance under constrained data availability. The proposed synthetic HAR dataset, including noise, fills a critical gap in the current literature by enabling the systematic evaluation of uncertainty-aware deep learning models under realistic noise conditions. Unlike traditional HAR datasets [34,35,36] focused on clean data and general activities, the dataset used in this work is processed to emulate constrained, safety-critical use cases, such as Fall and Cough detection in healthcare, offering unique value for robustness and interpretability studies. Table 1 summarizes the key differences between the proposed and existing HAR datasets.

In summary, this article contributes the following novelties to scientific research and the development of HAR prediction uncertainty analysis as follows:

- Most existing HAR studies optimize accuracy while overlooking uncertainty, and those that consider uncertainty usually apply only one estimation technique: MC Dropout or Bootstrap. This work provides a direct head-to-head evaluation of two fundamentally different paradigms of uncertainty estimation: stochastic regularization (MC Dropout) vs. ensemble resampling (Bootstrap). This systematic comparison clarifies their relative strengths, weaknesses, and trade-offs, which has not been explicitly addressed in the wearable HAR literature.
- Previous studies have concentrated on clean datasets, which limits real-world applicability. This work introduces controlled noise across accelerometer signals and evaluates how each uncertainty estimation method responds. This process adds practical value because healthcare and elderly care applications of HAR often operate in noisy, uncontrolled environments. The proposed system demonstrates how MC Dropout and Bootstrap handle degraded inputs, providing actionable insights for robust model deployment.
- While uncertainty estimation has been explored in other fields, such as brain–computer interfaces and radiation therapy, the HAR domain lacks guidelines on which approach to choose for wearable systems. By comparing the uncertainty sharpness, confidence intervals, and robustness to data constraints, our research offers a methodological framework for researchers and practitioners, supporting them in selecting the appropriate uncertainty method based on application needs, like computational cost vs. reliability in healthcare monitoring.

This research provides valuable insights into designing more robust HAR systems for health monitoring and medical diagnostics by systematically analyzing the trade-offs between computational complexity and prediction reliability. Unlike prior studies that focused primarily on accuracy improvement, this work systematically evaluates uncertainty estimation techniques, offering a new direction for enhancing the reliability of deep learning models in HAR.

The paper is organized as follows: The Section 2 describes the designed Deep Learning (DL) model with the data acquisition process. Then Section 3, the use of MC Dropout and Bootstrap in uncertainty estimation are described in detail. In the Section 4, data augmentation is presented, which can enhance the classification accuracy of NNs. The Section 5 presents the experiments, the results, the analysis, and conclusion.

## 2. Wearable Device and Deep Learning Model

### 2.1. Dataset

**Figure 2:**
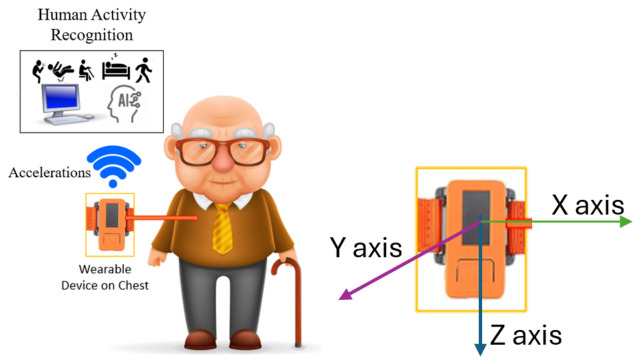
Wearable device on chest for HAR (**left**) and the 3-axis orientation of accelerometer (**right**).

As illustrated in Figure 2, an accelerometry-based wearable sensor (M5stickC) [37] was equipped on the user’s chest to acquire the acceleration data corresponding to 5 activity classes: class 0: Cough; class 1: Fall; class 2: Sit down; class 3: Sleep; and class 4: Walk. A 3-axis accelerometer was embedded in the wearable device, with 16-bit Analog-to-Digital Converters (ADCs) to sample each axis signal. The full-scale range was set at ±2000 mg for each of the X, Y and Z directions. A sampling frequency of 10 Hz was adopted because the dominant motion components associated with Cough and Fall activities occur within a low-frequency range and can be adequately captured according to the Nyquist criterion. In addition, the lower sampling rate reduces the computational and energy requirements, making it suitable for wearable healthcare-monitoring applications. Meanwhile, higher sampling frequencies increase the energy consumption and require more storage space for data. About 500,000 samples were acquired for each of the 5 activities. There were 5 participants in the study with an age range from 30 to 50 years old. 70% of the dataset was used for training and the rest was used for the testing process.

The three input features are the accelerations on X-, Y-, and Z-axes as shown in Figure 2, containing the following information:

- X acceleration (Xacc) represents the horizontal axis, aligned parallel to the shoulders for a left–right movement. If the user tilts their chest to the left, Xacc is negative. If the user tilts their chest to the right, Xacc is positive.
- Y acceleration (Yacc) represents the depth or forward–backward axis. If the user bends backward, Yacc is negative. If the user leans forward, Yacc is positive.
- Z acceleration (Zacc) represents the vertical axis, capturing movement along the up–down frame of the body. If the accelerometer detects an upward acceleration, Zacc is negative. If the accelerometer detects a downward acceleration, Zacc is positive.

The output event is decided by the class with the highest probability, given by the DL prediction.

**Device safety:** The prototype was implemented using an M5StickC PLUS2 development board (M5Stack Technology Co., Ltd., Shenzhen, China), which is a commercially available electronic device intended for consumer and educational applications. The M5StickC PLUS2 is a commercially available development platform certified for use in multiple regulatory jurisdictions. According to the manufacturer, the device carries CE (European Conformity), FCC (Federal Communications Commission), and Ministry of Internal Affairs and Communications (MIC) certifications, demonstrating compliance with applicable requirements for radio-frequency emissions, electromagnetic compatibility (EMC), and wireless communication equipment. In addition, the integrated rechargeable battery complies with IEC 62133, an international safety standard for portable rechargeable batteries. These certifications guarantee that the hardware has undergone regulatory conformity assessment for electrical, electromagnetic, wireless, and battery-related safety requirements.

- M5Stack Technology Co., Ltd. M5Stack Products Certification. Available online: https://docs.m5stack.com/en/learn/certification/certification (accessed on 20 February 2026).
- Federal Communications Commission (FCC). FCC ID: 2AN3WSTICKCPLUSV11—M5StickC PLUS2. Available online: https://fccid.io/2AN3WSTICKCPLUSV11 (accessed on 20 February 2026).

### 2.2. DL Model

The model is a DNN designed for multi-class classification with dropout applied at each hidden layer to estimate the uncertainty using MC Dropout during training and inference. The network consists of fully connected layers with customizable architecture and activation functions, using Softmax [38] for the final class prediction. The grid search method [39] is utilized to determine the most effective hyperparameters, such as the number of hidden layers, number of neurons, and dropout probability.

Layer 1: Input Layer

- The input to the network is defined as a vector of size equal to the number of features, which in this case is the total number of acceleration features along the three axes (X, Y, and Z).

Layer 2: Hidden Layers

- The hidden layers are fully connected, defined by the structure provided in the argument layers’ shape, which specifies the number of neurons in each layer.
- The first hidden layer is connected to the input layer, and the subsequent hidden layers are connected sequentially.
- The activation function used for all hidden layers is ReLU (Rectified Linear Unit) [40], which introduces non-linearity into the model, helping it learn complex patterns from the input data.
- Dropout is applied after each hidden layer to randomly deactivate a fraction of neurons during training (and test time in MC Dropout) to prevent overfitting and estimate uncertainty.
- The dropout probability controls the fraction of “dropped” neurons (set to zero) during training. This randomness helps generalize the model and creates diverse predictions for the same input during inference.

The network can contain multiple hidden layers. The for-loop dynamically builds each hidden layer and applies the dropout method.

In this case, the DL model structure is designed as follows:

- Input Layer: Processes the three input features and feeds them into the hidden layers.
- Hidden Layers: The network has three hidden layers to progressively extract features and learn patterns from the input data:
  ○ Hidden Layer 1 of 80 neurons.
  ○ Hidden Layer 2 of 40 neurons.
  ○ Hidden Layer 3 of 20 neurons.
- Activation function between hidden layers is ReLU.
- Output layer is configured with a Softmax activation function, which enables multi-class classification. Each output node represents one of the five activity classes, and the Softmax function converts the output to probabilities for each class. The activity with the highest probability is selected as the predicted class.

This model structure with progressively smaller hidden layers is designed to refine feature extraction as the data flows through the network, supporting accurate classification of each activity by minimizing the overfitting issue. The use of ReLU in the hidden layers ensures that the model can handle non-linear patterns. In contrast, the use of Softmax in the output layer allows for a clear probabilistic interpretation of each activity class, which is crucial for applications involving human activity recognition using wearable technology.

Layer 3: Output Layer:

The output layer uses a Softmax activation function, producing a probability distribution over the predicted classes. The Softmax function ensures that the outputs are non-negative and sum to 1, making them interpretable as class probabilities. Softmax essentially amplifies the difference between logits. A class with a slightly higher raw score gets a much higher probability. Classes with much lower scores get near-zero probability.

In the context of HAR, this probabilistic representation is particularly important for several reasons. Firstly, it enables the model to express the confidence levels associated with each predicted activity, which is essential when dealing with sensor noise, motion overlaps, or transitions between activities. Secondly, it allows for probabilistic decision-making, such as applying thresholds or smoothing techniques across consecutive time windows, to improve temporal consistency in recognition. Thirdly, the Softmax output forms the basis for computing the categorical cross-entropy loss during training, which measures the divergence between the predicted probability distribution and the true class label.

### 2.3. Monte Carlo Dropout Method

Once the network architecture is defined, the model is compiled with the appropriate loss functions and metrics. For multi-class classification, categorical cross-entropy is used as the loss function, and categorical accuracy is used to evaluate model performance during training [41]. During training, dropout is typically applied to prevent overfitting. However, MC Dropout introduces stochasticity into the predictions during inference by keeping the dropout layers active. This technique allows for multiple forward passes (predictions) for the same input, providing a way to estimate the uncertainty of the predictions. The dropout layers are active, simulating a form of ensemble averaging over different sub-networks. Dropout reduces overfitting by ensuring that the model does not rely too heavily on any single set of neurons.

By applying MC Dropout during inference, the model produces different outputs for the same input, which is used to compute the mean and variance of predictions. This method allows for uncertainty quantification, useful in cases where the model’s confidence is crucial. The model can be easily customized for different tasks by adjusting the layers’ shape, activation functions, and dropout rates.

The dropout probability in this model refers to the probability of “dropping”, or deactivating neurons in a layer during each forward pass. It is a hyperparameter that plays a crucial role in both regularization during training and uncertainty estimation during inference in this architecture. Dropout is a widely used regularization technique to prevent overfitting by randomly deactivating a fraction of neurons in each layer during training [42]. The dropout probability (also called dropout rate) determines how many neurons are dropped during each forward pass. Its working principle can be depicted as follows: Each neuron in a layer is randomly set to zero (dropped) with a configured probability. Only a subset of neurons contributes to the forward pass during training, making the network less reliant on specific neurons. For instance, if the dropout probability is set to 0.25, 25% of the neurons in a layer are randomly dropped during each forward pass, while the remaining 75% continue to contribute to the learning process.

By randomly deactivating neurons, dropout forces the network to learn more robust features. It prevents the model from becoming overly dependent on any single set of neurons, making the network less likely to overfit the training data. The more neurons are dropped, the stronger the regularization effect. However, if too many neurons are dropped, it can harm the model’s capacity to learn, especially if the network is shallow. Dropout is typically applied after each hidden layer to regularize the network. Generally, if the dropout probability is too low, such as 0.05, too few neurons are dropped during inference, and the model’s predictions across different passes will be similar, leading to low efficiency in uncertainty estimation. If the dropout probability is too high, such as 0.5 or above, many neurons will be dropped, and the network’s predictions will vary more across the forward passes, causing overestimated uncertainty. Typically, a moderate dropout probability, about 0.2 to 0.3, is used in practice to balance learning and uncertainty estimation.

In the tests, MC Dropout remains active during inference, simulating multiple different sub-networks during each forward pass by deactivating different neurons randomly.

- Multiple forward passes (predictions) are made for each input sample, and the dropout probability determines how many neurons are randomly deactivated in each pass.
- The randomness introduced by dropout during inference leads to variations in predictions for the same input. By making many predictions and analyzing the distribution of those predictions, we can estimate the mean (expected prediction) and the uncertainty (variance) in the predictions.

### 2.4. Bootstrap

The Bootstrap method is a statistical resampling technique that creates multiple resampled datasets from an original dataset. Each resample is generated by randomly selecting data points with replacement, meaning some points may be repeated, while others may not appear in the resampled dataset. For uncertainty estimation, the Bootstrap method involves the following steps.

Step 1: Bootstrap Samples Generation

Various DNN models with the same architecture are created for the bootstrap process. In this case, 50 models are utilized. Using 50 bootstrap-based DNN models provides a balanced compromise between statistical reliability and computational efficiency. Empirical and theoretical studies in ensemble learning and bootstrap estimation [43] show that beyond approximately 30–50 resamples, improvements in prediction stability and uncertainty estimation become marginal, while the computational costs increase significantly. Therefore, employing 50 models ensures that the ensemble captures sufficient variability from the training data to produce stable mean and variance estimates, leading to robust and reliable uncertainty quantification without requiring excessive training time or resource usage.

From the original training data, the bootstrap samples are generated. The system randomly picks samples from this dataset with replacements. If the training dataset has *L* samples, *L* samples will be randomly selected with replacement to create new bootstrap samples. This process is repeated to create multiple bootstrap samples. At the end of this procedure, each new DNN model will have *L* resampled samples.

Step 2: Model Training on Each Bootstrap Sample

Each DNN model is trained independently with its own bootstrap samples and learns slightly different *N* weights due to the differences in the training data it is exposed to. The network parameters vary, leading to slight differences in the prediction probabilities of each model. This introduces diversity in the predictions, which is crucial for uncertainty estimation.

Step 3: Predictions

After training all models, all the DNN models are evaluated by using the same test data. Each test sample has multiple predictions (one from each model). Since the models were trained on different subsets of the data, their predictions will vary.

For instance, the first test sample has predictions from 50 models, each providing a probability distribution across the 5 classes. These predictions may not all be identical, but they provide an idea of the consensus (mean) and disagreement (standard deviation) among the models.

Step 4: Mean and Standard Deviation Calculation

The predictions of the models will vary due to the different subsets of training data they were trained on. The mean of these predictions gives the most likely output, while the standard deviation quantifies the uncertainty (variance) in the predictions.

Unlike techniques as MC Dropout, Bootstrap does not require modification of the model architecture or keeping dropout active during inference. Instead, it involves resampling the training data to create multiple subsets and independently training a model on each. Since each model is exposed to a slightly different view of the data, the ensemble captures a range of plausible hypotheses. This diversity helps in quantifying the epistemic uncertainty, as disagreement among the models reflects uncertainty in regions where the training data is sparse or ambiguous. Additionally, when the input data is noisy or inconsistent, the ensemble may also reflect aleatoric uncertainty through variability in predictions, offering a more complete picture of the model’s confidence.

Here, epistemic uncertainty arises from limited knowledge or insufficient data and reflects the model’s uncertainty about its own parameters. Aleatoric uncertainty is caused by inherent noise or randomness in the observations and persists even with more data.

### 2.5. Calibration Metrics Evaluation

In DL models, calibration is an essential parameter, which shows that the predicted probability values from the classifier correspond to the true likelihood of being correct. It is not just about accuracy (whether predictions are correct), but about how trustworthy the confidence values are. To support the reliability evaluation of the proposed MC Dropout-based classifier, the calibration metrics are analyzed with Expected Calibration Error (ECE) [44] and Maximum Calibration Error (MCE) [45].

During classification, for each sample *i*:

- The model outputs the predicted probabilities *p_i_*(*c*) for each class *c* with *i* number of predictions.
- The confidence is defined as the maximum predicted probability:

(1) $$\mathrm{confidence}=\max\;p_{i}(c)$$

The ECE is the weighted average gap between confidence and accuracy across all bins. To compute the ECE, the confidence vs. accuracy is compared through the partitioning of the confidence values into intervals (bins). Each bin corresponds to a range of confidence scores.

With 10 bins and equal width = 0.1:

- Bin 1 → confidence in [0.0, 0.1);
- Bin 2 → confidence in [0.1, 0.2);
- …
- Bin 10 → confidence in [0.9, 1.0].

(2) $$ECE=\sum_{b=1}^{B}\frac{n_{b}}{N}|accuracy\left(b\right)-confidence\left(b\right)|$$

where *N* = total number of predictions.

*n_b_* = number of predictions that fall into bin b.

*B* = the number of bin.

Lower ECE is better (0 means perfect calibration).

MCE is the largest single-bin gap between confidence and accuracy. The MCE is more sensitive than the ECE because it highlights the worst-case calibration error.

### 2.6. Confidence Interval

The mean and standard deviation (St.Dev) across the specific number of predictions are calculated for each class in a sample. In each individual sample, the 95% confidence interval of the prediction probability is calculated as follows:

(3) $${\hat{p}}_{i}\pm 1.96\;\sigma_{i\;}$$

where

- ${\hat{p}}_{i}$ is the mean predicted probability for class *i*.
- *σ_i_* is the St.Dev of all predictions for class *i*.

### 2.7. Confidence Interval Uncertainty of Alerted Case with Each Input Feature

In the context of uncertainty estimation and model predictions, sharpness refers to a measure of the width of the predicted uncertainty interval, which reflects the confidence or certainty of the model in its predictions. It is commonly used when uncertainty is estimated through probabilistic models or techniques like MC Dropout and Bootstrap.

Sharpness is calculated by looking at the 95% confidence intervals around the predicted probabilities for a particular class, such as Cough or Fall, and measuring the average width of these intervals across different test samples.

Each input feature results in multiple predictions from the MC Dropout process or multiple Bootstrap models; so, for each instance, the sharpness is calculated as follows:

(4) $${LB}_{i}\;=\;{\hat{p}}_{i\;}-\;1.96\;\times \;\sigma_{i}$$

(5) $${UB}_{i}={\hat{p}}_{i}+1.96\;\times \;\sigma_{i}$$

(6) $$Sharpness=\sum_{i=1}^{K}{UB}_{i}-{LB}_{i}$$

where

- *LB* is Lower Bound;
- *UP* is Upper Bound;
- *K* is number of test samples.

## 3. Experiments and Results Analysis

### 3.1. DL Metric Evaluation

The evaluation metrics were analyzed for both the MC Dropout and Bootstrap techniques. In DL evaluation, accuracy reflects the overall proportion of correct predictions across all classes. Precision indicates the percentage of predicted positive instances that are truly positive, making it useful when the cost of false positives is high. Recall measures the percentage of actual positive instances that the model correctly identifies, which is important when missing positive cases is costly. The F1-score, defined as the harmonic mean of precision and recall, provides a balanced measure that is especially valuable for evaluating models on imbalanced datasets.

As reported in Table 2, Bootstrap consistently outperformed MC Dropout across multiple activity classes, yielding a 1% improvement in overall accuracy (91% vs. 90%) and more robust per-class performance. The most substantial improvement was observed in Fall detection, where the recall increased from 0.80 to 1.00. This improvement is particularly critical in health-monitoring applications, as undetected Falls can have severe consequences. For Cough and Sleep, Bootstrap achieved marginal but consistently superior results in both precision and recall, leading to higher F1-scores. The Sit-down activity showed a slight trade-off: while the recall decreased from 1.00 to 0.96, the precision improved from 0.91 to 0.95, resulting in a small net gain in the F1-score. Finally, the Walk activity benefited from Bootstrap with substantially better recall (+0.09), suggesting that this method better differentiates Walking from other activities.

In term of clinical implications, both MC Dropout and Bootstrap achieved high classification performance, with overall accuracies of 90% and 91%, respectively. For Cough detection, both methods obtained high recall (94%), indicating that most Cough events were successfully identified. Although some false-positive Cough detections were observed, these errors mainly resulted in unnecessary alerts rather than missed health-related events. A more clinically significant difference was observed for Fall detection. While both methods achieved perfect precision (1.00), MC Dropout achieved a recall of 0.80, whereas Bootstrap achieved a recall of 1.00. This indicates that MC Dropout failed to detect some Fall events, while Bootstrap successfully identified all Falls in the dataset. In healthcare and elderly care applications, missed Falls (false negatives) are particularly critical because they may delay assistance and increase the risk of serious injury. Therefore, the higher Fall recall achieved by Bootstrap suggests improved clinical reliability and safety for real-world monitoring applications. Practically, the comparative analysis demonstrates that Bootstrap uncertainty estimation provides more reliable classification performance than MC Dropout, with clear advantages in high-stakes activities, such as Fall detection. This metric comparison highlights its suitability for safety-critical applications in activity recognition and healthcare monitoring.

Moreover, the *t*-test and the *p*-value are employed to assess whether the observed differences in model accuracy between the MC Dropout and Bootstrap methods were statistically significant. To carry out the procedure, the dataset is split into 10 equal folds. In each iteration, nine folds are used for training, and one fold is used for testing. This process repeats 10 times, each time with a different fold as the test set.

Using the formula

(7) $$t=\frac{\bar{d}}{std/\sqrt{n}}$$

where $\bar{d}$ is the mean of differences between each fold, std is the standard deviation, and *n* is the number of folds:

If *t* > 0: MC Dropout tends to perform better than Bootstrap (since differences are positive).

If *t* < 0: Bootstrap tends to perform better.

The *p*-value is the probability of observing a t-statistic as extreme (or more extreme) than *t*, under the null hypothesis that *μd* = 0, where the true mean difference between paired observations is zero.

(8) $$p=2\;P(T_{df=n-1}\geq \left|t\right|)$$

where $T_{df=n-1}$ follows a Student’s t-distribution with n − 1 degrees of freedom (df):

*p* < 0.05 → statistically significant (conventional level).

*p* < 0.01→ highly significant.

*p* < 0.001→ very highly significant.

*p* > 0.05 → fail to reject the null hypothesis (not statistically significant—the difference could just be due to random variation).

The obtained results from the accuracy analysis of the two methods are as follows: paired *t*-test: *t* = −6.1515, *p* = 0.0035. Thus, Bootstrap achieved a higher mean accuracy compared to MC Dropout. Since *p* < 0.05, the difference is highly statistically significant. The small *p*-value means that the stronger the evidence against the null hypothesis, the less likely the observed difference is due to random chance.

### 3.2. Uncertainty Visualization and Analysis

**Figure 3:**
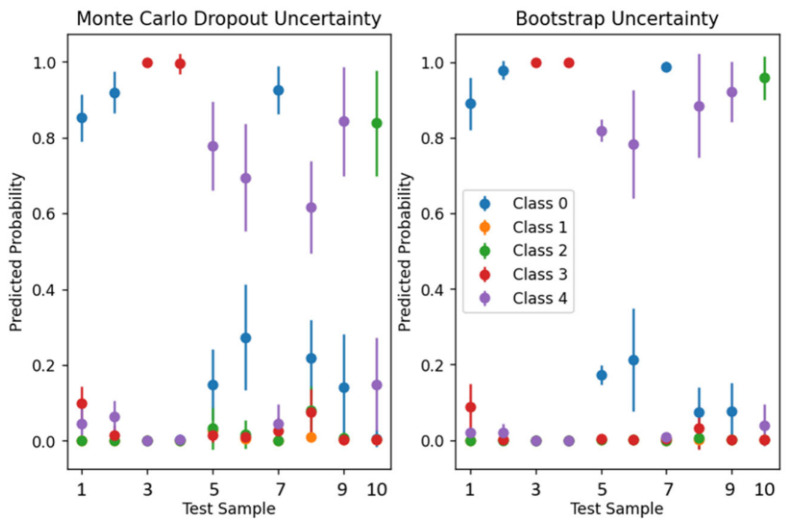
Uncertainty visualization of MC Dropout and Bootstrap models.

As shown in Figure 3, each point on the plot represents the mean predicted probability of a given class, ${\hat{p}}_{i\;}$, for a specific test sample. The error bars around each point represent that prediction’s uncertainty (St.Dev $\sigma_{i}$). This uncertainty is derived for MC Dropout, where multiple predictions are made for each test sample, and the St.Dev of these predictions gives an estimate of uncertainty. There are also cases where all the probability predictions of 50 forward passes are the same. For instance, test sample 2 in both the MC Dropout and Bootstrap charts has “zero uncertainty” because the DL model produced the same predictions, categorizing a certain output as class 3. For the 10 samples shown, the Bootstrap method produced prediction results with less uncertainty than MC Dropout.

These charts provide a detailed view of the model’s confidence and uncertainty for each test sample across all classes. Examining these predictions and uncertainties makes it possible to identify cases where the model is highly uncertain (long error bars) and may need improvement.

### 3.3. Calibration Metrics

In this research, the calibration analysis is intentionally concentrated on the Cough and Fall activity classes because these two events are the most safety critical and clinically relevant among the five considered classes (Sit, Sleep, Walk, Cough, and Fall). Unlike the remaining daily activities, Cough and Fall represent high-risk events where incorrect or poorly calibrated predictions can have direct consequences for user safety and intervention timing. For instance, Fall detection models must avoid overconfident misclassifications, as failing to identify a true Fall can lead to delayed assistance, while Cough detection is associated with early symptom monitoring and health assessment. Therefore, understanding not only the accuracy but also the reliability and trustworthiness of predicted probabilities is essential for these two classes.

For the Cough class, the calibration analysis yielded an ECE of 0.0383 and an MCE of 0.1691. The relatively low ECE indicates that the predicted probabilities for Cough events are well aligned with the true occurrence frequencies. The modest MCE value further suggests that there are no severe local regions of miscalibration, making the model’s probability estimates for this class generally reliable.

For the Fall class, the model achieved an even lower ECE of 0.0103, suggesting near-perfect average calibration. However, the MCE of 0.2418 reveals that, in some confidence regions, the model still exhibits noticeable miscalibration, likely due to localized overconfidence or under confidence. This is particularly relevant given the high-risk nature of Fall detection, where overconfident but incorrect predictions could reduce system trustworthiness.

Taken together, these results indicate that the model provides reliable probability estimates overall for both Cough and Fall, with the Fall predictions being especially well calibrated on average. Nevertheless, the elevated MCE for Fall underscores the importance of addressing localized miscalibration, as probability reliability is critical in applications where safety-related interventions may depend on model outputs.

**Figure 4:**
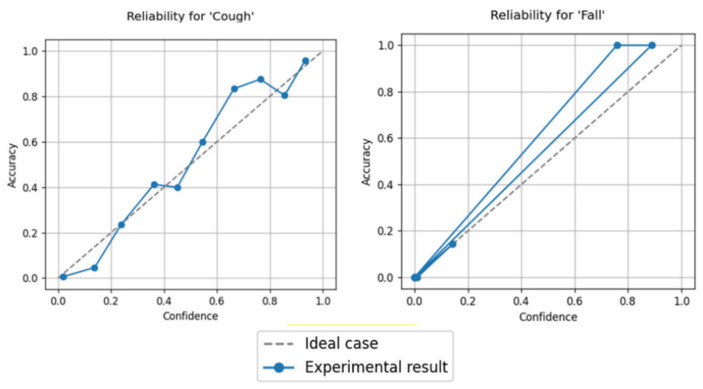
Reliability diagram.

As shown in Figure 4, the reliability diagrams demonstrate a positive correlation between prediction confidence and accuracy, indicating that higher-confidence predictions are more likely to be correct in the Cough case. This behavior is desirable, as it suggests that a prediction made with high confidence corresponds to a higher empirical accuracy, whereas lower-confidence predictions are less reliable. In the Fall activity class, the reliability diagram shows that multiple confidence bins, such as 0.7 and 0.9, can achieve an accuracy of 1.0, indicating that the predictions within both the medium- and high-confidence ranges are perfectly correct. This point reflects strong calibration and reliable probability estimates for Fall detection. This trend aligns with the fundamental goal of calibration, which is to ensure that confidence values are trustworthy. For instance, a model that outputs 80% confidence should be correct approximately 80% of the time. The observed relationship between confidence and accuracy in our results confirms that the classifier is reasonably well calibrated, with deviations captured quantitatively by the ECE and MCE.

As reported in Table 3, MC Dropout achieved a lower ECE for the Cough class, indicating that its average confidence values were more aligned with the observed accuracies, and had better overall calibration. However, Bootstrap exhibited a lower MCE, meaning its worst-case miscalibration (largest gap between confidence and accuracy across bins) was smaller. Thus, while MC Dropout provided more consistent calibration on average, Bootstrap avoided extreme calibration errors.

For the Fall class, MC Dropout shows a very low ECE (0.0103), suggesting it is almost perfectly calibrated on average. However, it suffers from a higher MCE (0.2418), indicating that in some confidence ranges the predictions deviate significantly from true accuracy. On the other hand, Bootstrap shows a higher ECE, but its MCE is much lower, implying it sacrifices average calibration quality to ensure more stable calibration across all bins.

### 3.4. Uncertainty of Alerted Case with Each Input Feature

Synthetic noise was introduced during the uncertainty-guided data augmentation stage. For Monte Carlo Dropout, high-uncertainty samples were first identified from multiple stochastic forward passes, and Gaussian perturbations were then added to these samples to generate synthetic training instances. For Bootstrap augmentation, high-uncertainty samples were randomly resampled with replacement and subsequently perturbed using Gaussian noise. In both cases, the injected noise followed a zero-mean Gaussian distribution with a standard deviation of 10% in the normalized feature space. In real-world deployments, accelerometer measurements are affected by multiple sources of disturbance, including sensor electronic noise, device displacement, body movement artifacts, variations in sensor placement, and environmental influences. Gaussian noise is highly effective in sensor signal processing as a representative model for these stochastic perturbations.

**Figure 5:**
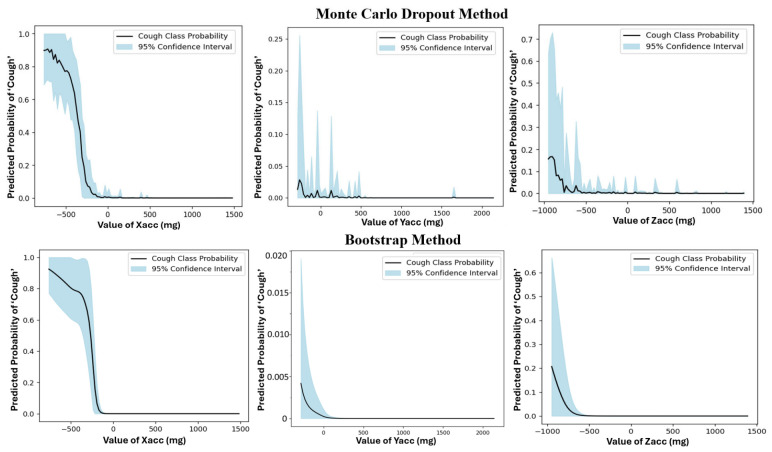
Uncertainty of each feature with respect to Cough class, based on minimum to maximum value of acquired acceleration for each case.

**Figure 6:**
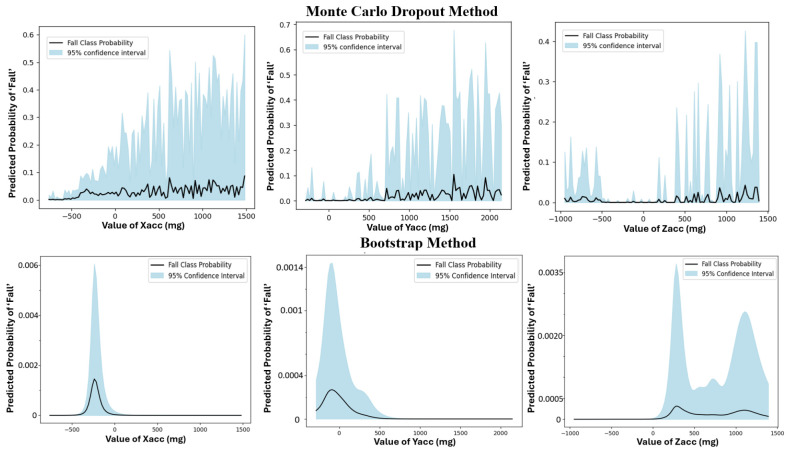
Uncertainty of each feature with respect to Fall class, based on minimum to maximum value of acquired acceleration for each case.

Figure 5 and Figure 6 show the uncertainty of Fall and Cough predictions, respectively, for each acceleration parameter. In the case of Cough uncertainty, both MC Dropout and Bootstrap methods demonstrate high fluctuations in the first interval value of Xacc, [−800 −50] mg. This suggests that extreme or highly negative *X*-axis accelerations are less frequently represented or more variable in the data, leading the model to be less confident and to assign broader predictive distributions. Bootstrap has null values after that interval, while MC Dropout still has minor uncertainty later, indicating that Bootstrap tends to collapse to very confident predictions once it moves away from these extreme values, whereas MC Dropout preserves some residual epistemic uncertainty even in more typical Xacc ranges. This behavior implies that MC Dropout may be more sensitive to subtle local variations in a signal, while Bootstrap behaves more decisively, which can be beneficial or risky depending on whether those regions correspond to true Cough events or to ambiguous motion patterns.

For the Yacc value, the prediction uncertainty is significant at intervals of [−180 −140] mg, [−20 −5] mg, and [100 118] mg for MC Dropout. These intervals likely correspond to specific motion phases, like transitions or small directional changes, where Cough and non-Cough activities can produce partially overlapping *Y*-axis patterns, making the discrimination task inherently more difficult. Bootstrap has the highest uncertainty at the minimum value of Yacc, then gradually decreases to 8 mg as the ending point of uncertainty. This indicates that, for Bootstrap, the most critical region is around extreme negative Yacc values, after which the model becomes increasingly confident. From a practical standpoint, these Yacc intervals can be interpreted as risk zones where the model should be monitored more carefully, thresholds may need refinement, or additional features (e.g., temporal context or multi-axis fusion) should be emphasized to reduce misclassification risk.

Regarding Zacc, prediction uncertainties of MC Dropout and Bootstrap also have peaks at the minimum values of the acceleration, then go down and end at −450 mg for Bootstrap. This again highlights that very low *Z*-axis accelerations are associated with higher ambiguity, possibly reflecting uncommon or boundary motion patterns in the training data. Meanwhile, MC Dropout still shows a prediction probability variation in some other intervals, including positive values such as [100 120] mg and [580 600] mg. The presence of uncertainty in these positive Zacc ranges suggests that, even when the vertical acceleration is relatively high, there exist motion patterns for which the model cannot fully distinguish between Cough and other activities. Consequently, these Zacc intervals identify regions where the model’s predictions should be interpreted with caution and where design choices, such as additional sensors or more advanced post-processing rules, may help to improve robustness of outcomes.

In the case of Fall uncertainty, the prediction uncertainty is more significant than in the Cough class, especially for MC Dropout, which exhibits a general trend of increasing uncertainty as the Xacc value rises. This is particularly evident in the Xacc intervals of [600–1500] mg, as well as the Yacc intervals of [1500–1800] mg and [1800–2200] mg, and to a lesser extent in the Zacc interval around [1200–1300] mg. Although the Zacc uncertainty is relatively small compared with that of Xacc and Yacc, these patterns indicate that high-magnitude accelerations, corresponding to impact-like or abrupt movements characteristic of Falls, produce greater epistemic uncertainty. This occurs because such extreme values are less frequent, more variable, and more difficult for the model to generalize, particularly under noise perturbation. As a consequence, the MC Dropout model expresses reduced confidence precisely in the acceleration regions most associated with high-risk events, reflecting the underlying difficulty of distinguishing true Falls from other strong, rapid movements, such as stumbling, collision, or sudden posture changes.

The Bootstrap method shows a more localized distribution of uncertainty across specific acceleration intervals. For Xacc, the uncertainty is concentrated within [−300–10] mg; for Yacc, within [−180–530] mg; and for Zacc, within [0–1500] mg. Beyond these ranges, the predicted probability variation collapses to zero, meaning that Bootstrap becomes extremely confident, sometimes overly confident, outside its uncertainty zones. This narrower uncertainty band suggests that Bootstrap relies more heavily on the regions where the training samples cluster, resulting in sharp transitions between uncertain and fully confident predictions. While this behavior can simplify interpretation, it may also imply reduced sensitivity to rare or atypical Fall-like patterns. Consequently, Bootstrap is able to perform well when acceleration signals stay within typical ranges but may fail to express meaningful uncertainty in rare high-impact events, which are precisely the scenarios where uncertainty estimation is most valuable in a safety-critical system.

Overall, this analysis demonstrates that MC Dropout is more sensitive to variations in input features, particularly within specific intervals, leading to fluctuating uncertainty. Bootstrap tends to have more localized uncertainty and a more stable distribution, with uncertainty dropping off after a certain interval. The sharper uncertainty spikes in MC Dropout suggest the model’s heightened sensitivity to certain input variations, while Bootstrap tends to be more conservative in modeling uncertainty outside defined intervals.

### 3.5. Sharpness Evaluation

As reported in Table 4, MC Dropout processes the Xacc and Zacc with relatively similar sharpness values in the Cough class, around 0.1 for Xacc and 0.0676 for Zacc. This process suggests a moderate fluctuation in predictions with respect to Xacc and Zacc. The Yacc sharpness in MC Dropout is significantly smaller (0.0132), indicating low uncertainty in the predictions across different Yacc values for the Cough class. In contrast, Xacc shows significantly higher sharpness in MC (0.2209) than in Bootstrap (0.0003) in the Fall class.

For MC Dropout, this suggests greater uncertainty in its predictions as the *X*-axis acceleration varies within the Fall class. The Yacc and Zacc sharpness in MC Dropout for Fall also show moderate uncertainty, with values of 0.1745 and 0.0727, respectively, though these values are still higher than those of Cough.

For the Bootstrap Method, Xacc and Yacc have significantly lower sharpness values for Fall (0.0003 and 0.0002, respectively), showing very stable predictions for these features in the Bootstrap method. The Zacc sharpness in Bootstrap for both classes is relatively low, with values of 0.0392 for Cough and 0.0008 for Fall, suggesting that the uncertainty related to Zacc is quite minimal in Bootstrap.

Practically, MC Dropout shows higher uncertainty (larger sharpness) for the Fall class, especially for Xacc and Yacc. Bootstrap shows much lower uncertainty for both classes across all features, particularly for Fall, where the sharpness values are close to zero, indicating a much more stable model under the Bootstrap method.

**Figure 7:**
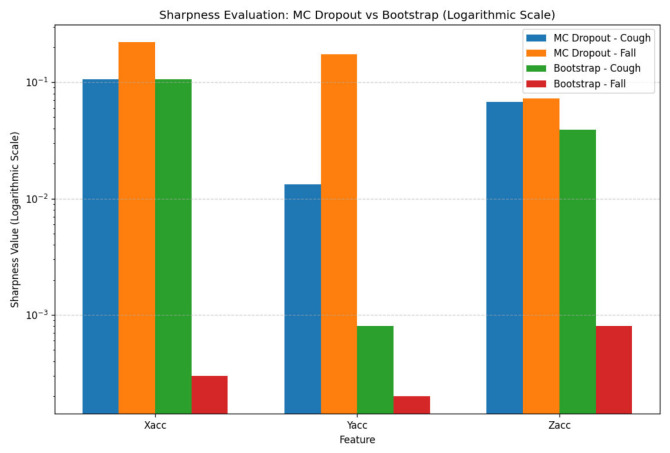
Sharpness values of MC Dropout and Bootstrap methods.

Figure 7 shows a visual comparison of the sharpness values of MC Dropout and Bootstrap methods across the acceleration features (Xacc, Yacc, and Zacc) for both Cough and Fall classes. The chart is plotted in logarithmic scale, so small values become visible and large values are compressed, enabling fair comparison. The results show the following characteristics:

- MC Dropout has noticeably higher sharpness with greater uncertainty than Bootstrap, especially for the Fall class.
- Bootstrap yields much lower sharpness across all features, indicating more stable and confident predictions, particularly for the Fall class, where uncertainty is minimal.

### 3.6. Computation Cost

The developed systems were operated by a 13th Gen Intel(R) Core(TM) i9-13900H (2.60 GHz) PC processor with RAM of 64.0 GB. The DL model was saved in TensorFlow Lite (TFLite) format, so each model size was evaluated.

As reported in Table 5, each Bootstrap model had a faster training time than MC Dropout. However, more Bootstrap models were involved, so a longer training time was required. Each Bootstrap model had a lighter size than MC Dropout. To deploy the edge device, it was possible to train many Bootstrap models, then select the best-performing one.

### 3.7. Overall Comparison

Table 6 demonstrates that while MC Dropout excels at capturing fine-grained uncertainty variations, it may introduce higher noise or false alerts in some intervals. On the other hand, Bootstrap provides a robust and stable uncertainty estimation, especially useful in mission-critical scenarios like Fall detection where reliability is paramount.

### 3.8. Sharpness Evaluation on External Dataset

The dataset from Wireless Sensor Data Mining Lab [46] includes accelerometer data from smart phones for activity recognition. The dataset consists of three accelerometer features (Xacc, Yacc, and Zacc) with the labelled activities. In this case, 100,000 samples of Sitting and Walking were randomly extracted from 29 volunteers who carried a smart phone. These data were used as external datasets for further testing the sharpness evaluation techniques of the system developed in this paper. The large number of participants allowed the system to be tested with more diverse data, which was very suitable for providing further analysis and performance verification of the proposed approach.

**Figure 8:**
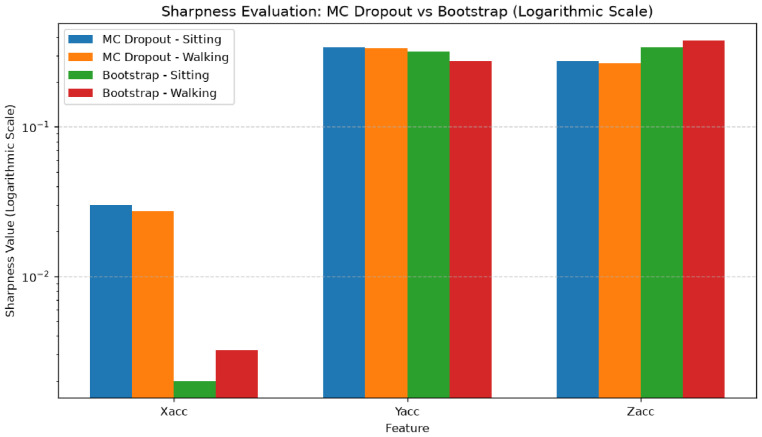
Sharpness values of MC Dropout and Bootstrap methods on WISDM dataset.

Table 7 and Figure 8 present the sharpness values obtained using Monte Carlo Dropout (MCD) and Bootstrap uncertainty estimations for Sitting and Walking activities based on acceleration. The results demonstrated notable differences between the two uncertainty estimation techniques. For the *X*-axis acceleration (Xacc), Bootstrap produced substantially lower sharpness values (0.0020 for Sitting and 0.0032 for Walking) compared with Monte Carlo Dropout (0.0301 and 0.0272, respectively). This point indicates that the predictions associated with Xacc remained highly stable across the different bootstrap resamples of the training data, suggesting that Xacc provides strong discriminatory information between Sitting and Walking. In contrast, Monte Carlo Dropout yielded wider confidence intervals due to the stochastic perturbation of network weights during inference, resulting in a more conservative estimate of uncertainty.

For the *Y*-axis acceleration (Yacc), both methods reported comparatively large sharpness values. Monte Carlo Dropout produced sharpness values of 0.3397 and 0.3368 for Sitting and Walking, respectively, while Bootstrap yielded values of 0.3183 and 0.2747. The relatively close agreement between the two approaches suggests that the uncertainty associated with Yacc is intrinsic to the feature itself rather than arising from model parameter variability. In other words, Yacc appears to contain less discriminative information for distinguishing between the two activities, leading both uncertainty estimation methods to assign broader confidence intervals.

A different trend was observed for the *Z*-axis acceleration (Zacc). Bootstrap produced substantially higher sharpness values (0.3399 for Sitting and 0.3785 for Walking) than Monte Carlo Dropout (0.2762 and 0.2669, respectively). This result suggests that the uncertainty associated with Zacc is strongly influenced by the composition of the training dataset. Since Bootstrap explicitly measures the sensitivity of model predictions to variations in the training samples, the wider confidence intervals indicate that the Zacc-related predictions were more affected by sampling variability than by uncertainty in the learned network parameters. Consequently, Bootstrap identified a larger epistemic uncertainty component for this feature.

An additional observation concerns the consistency between activity classes. Monte Carlo Dropout produced remarkably similar sharpness values for Sitting and Walking across all three features, indicating that the uncertainty estimates are largely independent of class membership. In contrast, Bootstrap exhibited greater variation between classes, particularly for Yacc and Zacc. This behavior suggests that Bootstrap is more sensitive to class-specific variations in the underlying data distribution and may therefore capture dataset-induced uncertainty more effectively.

Overall, based on the proposed approach, the results indicate that the two uncertainty estimation techniques capture different aspects of predictive uncertainty. Monte Carlo Dropout primarily reflects uncertainty associated with the model’s parameters and tends to produce smoother and more homogeneous uncertainty estimates. Bootstrap, on the other hand, is more sensitive to variability in the training data and therefore provides a stronger indication of dataset-dependent uncertainty. The agreement between both methods for Yacc strengthens the conclusion that this feature contributes the highest intrinsic ambiguity to the classification task. Conversely, the divergence observed for Xacc and Zacc highlights the importance of employing multiple uncertainty estimation techniques when analyzing wearable sensor data, as different methods may reveal complementary sources of uncertainty.

## 4. Discussion

This study presents a comprehensive comparison of two widely adopted uncertainty estimation techniques, MC Dropout and Bootstrap, in deep learning-based human activity recognition (HAR) using wearable sensor data. While previous HAR research has predominantly focused on improving classification accuracy, the present work emphasizes the importance of uncertainty quantification as a critical component for the trustworthy and reliable deployment of deep learning models. To support a rigorous evaluation, the proposed PUAF integrates traditional classification metrics with statistical significance testing, uncertainty visualization, calibration measures (ECE and MCE), confidence interval analysis, confidence interval sharpness, and computational cost assessment. This framework enables a multidimensional evaluation of uncertainty-aware models, extending beyond predictive accuracy to include reliability, calibration quality, and practical deployment considerations. The experimental results demonstrate that both uncertainty estimation methods improve model robustness, particularly under noisy sensor conditions where random variations in accelerometer signals can adversely affect classification performance. The noise injection experiments provide a realistic assessment of model behavior under uncertainty and highlight the distinct characteristics of the two approaches. The sharpness analysis demonstrates that MC Dropout and Bootstrap capture complementary aspects of predictive uncertainty. On the original HAR dataset, MCD consistently produced higher sharpness values than Bootstrap, particularly for the Fall class, indicating greater sensitivity to uncertainty in the learned model parameters. This behavior suggests that Fall-related motion patterns introduce higher epistemic uncertainty, causing prediction variability when network weights are stochastically perturbed during inference. In contrast, Bootstrap yielded near-zero sharpness values for the Fall class across all acceleration axes, implying that its predictions remained highly stable under variations in the training samples. This finding indicates that the data-driven uncertainty was relatively low and that the available training data adequately represented Fall-related patterns. For the Cough class, both methods produced lower sharpness values, suggesting a more homogeneous activity distribution and reduced uncertainty.

The present work employed a sample-level train/test split, which may have allowed samples from the same participant to appear in both training and testing sets. As commonly recognized in HAR research, this evaluation protocol may partially capture subject-specific characteristics and therefore yield a higher classification performance than strictly subject-independent validation schemes. However, the main objective of this work was to investigate and compare uncertainty estimation techniques in Neural Networks rather than to establish the generalization performance of a specific activity recognition model. To further assess the robustness of the proposed uncertainty analysis framework, an additional evaluation was conducted using the external WISDM dataset, which contains data collected from 29 independent participants. Although the external dataset did not include the target Cough and Fall activities, it provided a substantially different activity recognition scenario involving Sitting and Walking activities from a larger and more diverse subject population. The external WISDM dataset revealed feature-dependent uncertainty characteristics. For Xacc, Bootstrap produced substantially lower sharpness than MC Dropout, indicating that Xacc provides robust discriminative information with minimal dataset-induced uncertainty. Conversely, both methods reported similarly high sharpness for Yacc, suggesting that the uncertainty originates primarily from intrinsic feature ambiguity rather than model or data variability. For Zacc, Bootstrap generated higher sharpness values than MCD, demonstrating stronger sensitivity to the training data composition and highlighting the presence of dataset-dependent epistemic uncertainty. These results obtained using this dataset demonstrate consistent and interpretable uncertainty behavior for both the Monte Carlo Dropout and Bootstrap methods, confirming their ability to capture complementary sources of predictive uncertainty. Therefore, the external validation supports the reliability and applicability of the proposed uncertainty analysis approach beyond the specific activities and participants in the original dataset. Practically, MC Dropout mainly reflects uncertainty associated with model parameters, whereas Bootstrap is more sensitive to uncertainty arising from training data variability. The agreement between both methods for Yacc and their divergence for Xacc and Zacc indicate that different accelerometer features are influenced by distinct uncertainty sources. These outcomes emphasize the importance of employing multiple uncertainty estimation techniques to obtain a comprehensive assessment of model reliability in human activity recognition systems.

On the other hand, although this study focused on Monte Carlo Dropout and Bootstrap uncertainty estimation methods implemented using a feed-forward Neural Network architecture, other state-of-the-art uncertainty quantification approaches, such as Deep Ensembles, Bayesian Neural Networks, and evidential deep learning, as well as more advanced HAR architectures, including CNNs, LSTMs, GRUs, and Transformer-based models, have demonstrated promising performance in related applications. These methods were not included in the present work because the primary objective was to compare two representative and computationally practical uncertainty estimation paradigms under controlled noisy conditions using a common baseline architecture. This design enabled a fair assessment of uncertainty estimation behavior while minimizing the confounding effects arising from architectural differences. Future research will extend the proposed framework to incorporate additional uncertainty quantification techniques and advanced deep learning architectures, and will investigate their relative performance, computational requirements, uncertainty characteristics, and robustness under varying noise conditions.

## 5. Conclusions

This work provides an in-depth analysis of uncertainty estimation in deep learning-based HAR by systematically comparing MC Dropout and Bootstrap-based approaches using wearable sensor data. Beyond classification performance, the study introduces the PUAF system, which combines predictive metrics, statistical testing, calibration analysis, uncertainty visualization, confidence interval evaluation, and computational cost assessment into a unified evaluation methodology.

The results demonstrate that both uncertainty estimation techniques enhance model robustness and provide meaningful confidence information under noisy conditions. MC Dropout captures detailed variations in predictive confidence and offers valuable insights into feature sensitivity, whereas Bootstrap produces more stable, conservative, and well-calibrated uncertainty estimates. These complementary characteristics highlight the importance of considering both predictive performance and uncertainty behavior when designing reliable HAR systems.

The contributions of this work extend beyond performance improvement by establishing a structured framework for uncertainty-aware HAR evaluation and providing practical recommendations for selecting uncertainty quantification methods in wearable-based health-monitoring applications.

Future work will investigate these approaches on larger and more diverse datasets to further assess classification performance and uncertainty reliability through metrics such as ECE, MCE, and reliability diagrams. Additionally, hybrid uncertainty estimation strategies that combine the strengths of MC Dropout and Bootstrap will be explored. Future studies will also extend uncertainty-aware learning to additional sensor modalities, including gyroscopes and physiological signals, and evaluate alternative uncertainty quantification methods to further advance robust and trustworthy HAR systems.

## Figures and Tables

**Table 1:** Comparison of the proposed and existing HAR datasets.

Aspect	Standard HAR Datasets	Proposed Synthetic HAR Dataset
Sensor Noise	Typically, minimal or real-world background noise; not explicitly modeled	Systematically injected noise to simulate varying real-world conditions
Uncertainty Focus	Does not quantify or label uncertainty; the focus is on accuracy	Designed to evaluate model uncertainty (aleatoric + epistemic) under noisy conditions
Context Simulation	Static, limited environments (e.g., lab-based setups)	Noise and variations simulate dynamic, real-world variability and sensor degradation
Evaluation Challenge	Mostly used for classification benchmarking	Built to stress-test model robustness and uncertainty estimation methods
Labeling Approach	Manual annotation or sensor timestamp-based segmentation	Ground truth is inherited from real data, then perturbed to generate synthetic realism
Data Diversity	Often lacks edge-case samples or rare events	Includes rare but critical activities and simulates scarce data conditions

**Table 2:** Classification report of DL models.

Class	Metric	MC Dropout	Bootstrap
Cough	Precision	0.86	0.88
	Recall	0.94	0.94
	F1-Score	0.90	0.91
Fall	Precision	1.00	1.00
	Recall	0.80	1.00
	F1-Score	0.89	1.00
Sit Down	Precision	0.91	0.95
	Recall	1.00	0.96
	F1-Score	0.95	0.96
Sleep	Precision	0.96	0.98
	Recall	0.80	0.84
	F1-Score	0.87	0.91
Walk	Precision	0.85	0.87
	Recall	0.80	0.89
	F1-Score	0.83	0.88
Overall Accuracy	–	90%	91%

**Table 3:** Comparison of calibration metrics between the two methods.

Parameter	MC Dropout	Bootstrap
Cough ECE	0.0383	0.0876
Cough MCE	0.1691	0.1303
Fall ECE	0.0103	0.0807
Fall MCE	0.2418	0.1092

**Table 4:** Sharpness evaluation.

	Monte Carlo Dropout		Bootstrap	
Feature	Cough Sharpness	Fall Sharpness	Cough Sharpness	Fall Sharpness
Xacc	0.1066	0.2209	0.1055	0.0003
Yacc	0.0132	0.1745	0.0008	0.0002
Zacc	0.0676	0.0727	0.0392	0.0008

**Table 5:** Computation cost report.

Parameter	MC Dropout	Bootstrap
Training time	975 s	292 s per Bootstrap model
Model size of 32-bit floating point (FP32)	4 MB	2 MB per each model
Model size of 8-bit integers (INT8)	1 MB	0.5 MB per each model

**Table 6:** Overall comparison of MC method and Bootstrap method.

Aspect	Monte Carlo Dropout	Bootstrap
Method Type	Stochastic regularization via dropout at inference (Bayesian approximation)	Resampling-based ensemble method (frequentist uncertainty estimation)
Uncertainty Estimation	Based on prediction variance across multiple dropout-enabled forward passes	Based on variation across multiple models trained on bootstrapped datasets
Prediction Stability	Less stable; sensitive to feature variations; more fluctuations in predictions	More stable; lower fluctuations; localized uncertainty
Sensitivity to Input Features	High sensitivity; uncertainty varies sharply across intervals (esp. for Fall class)	Conservative sensitivity; uncertainty concentrated in specific input intervals
Calibration metrics	Lower ECE; better overall calibration	Lower MCE; minimizes extreme calibration errors
Sharpness (Cough Class)	Moderate: Xacc (0.1066), Yacc (0.0132), Zacc (0.0676)	Low: Xacc (0.1055), Yacc (0.0008), Zacc (0.0392)
Sharpness (Fall Class)	High sharpness in Xacc (0.2209) and Yacc (0.1745); moderate in Zacc (0.0727)	Very low sharpness in Xacc (0.0003) and Yacc (0.0002); minimal in Zacc (0.0008)
Uncertainty Distribution	Widespread across multiple value ranges; continues beyond peak intervals	Localized in specific acceleration intervals; almost no uncertainty outside them
Prediction Certainty	Zero uncertainty observed in repeated predictions (identical outputs across passes)	Same certainty observed for certain samples with consistent predictions
Best-Use Case	Exploratory analysis of feature impact and model sensitivity	Stable deployment with bounded uncertainty in critical applications
Interpretability	Shows sharp spikes in uncertainty for interpretation of sensitive intervals	Easier to interpret due to smoother and confined uncertainty regions

**Table 7:** Sharpness evaluation on External data.

	Monte Carlo Dropout		Bootstrap	
Feature	Sitting Sharpness	Walking Sharpness	Sitting Sharpness	Walking Sharpness
Xacc	0.0301	0.0272	0.0020	0.0032
Yacc	0.3397	0.3368	0.3183	0.2747
Zacc	0.2762	0.2669	0.3399	0.3785

## Data Availability

The data presented in this study are available on request from the corresponding author due to privacy.
